# A Redox-Silent Analogue of Tocotrienol May Break the Homeostasis of Proteasomes in Human Malignant Mesothelioma Cells by Inhibiting STAT3 and NRF1

**DOI:** 10.3390/ijms23052655

**Published:** 2022-02-28

**Authors:** Kyota Ishii, Momoka Fusegi, Tatsuki Mori, Kosuke Teshima, Nanako Ninomiya, Kakeru Kohno, Ayami Sato, Tatsuya Ishida, Yuichi Miyakoshi, Tomohiro Yano

**Affiliations:** 1 Laboratory of Molecular Bromacology, Graduate School of Food and Nutritional Sciences, Toyo University, Oura District, Gunma, Itakura Town 374-0193, Japan; s3c102100017@toyo.jp (K.I.); s1c111400434@gmail.com (M.F.); 2 Department of Food and Life Sciences, Faculty of Food and Nutritional Sciences, Toyo University, Oura District, Gunma, Itakura Town 374-0193, Japan; s1C121900306@toyo.jp (T.M.); s1C121900317@toyo.jp (K.T.); s1C121800248@toyo.jp (N.N.); 3 Research Institute of Life Innovation, Toyo University, Oura District, Gunma, Itakura Town 374-0193, Japan; k-kouno@dokkyomed.ac.jp (K.K.); otas.imaya@gmail.com (A.S.); tatsu_34@qb4.so-net.ne.jp (T.I.); miyakoshi@toyo.jp (Y.M.)

**Keywords:** cytotoxicity, ER stress, malignant mesothelioma cells, NRF1, proteasome inhibitor, redox-silent analogue, STAT3, tocotrienol

## Abstract

6-*O*-Carboxypropyl-alpha-tocotrienol (α-T3E) is a multi-target redox-silent analogue of tocotrienol that exhibits cytotoxicity against many cancer cells, including malignant mesothelioma (MM) cells. α-T3E has several molecular targets to effectively induce cytotoxicity against MM cells; however, the mechanisms underlying this cytotoxicity remain unclear. In the present study, we demonstrated that the α-T3E-dependent disruption of the homeostasis of proteasomes strongly induced endoplasmic reticulum (ER) stress, which resulted in effective cytotoxicity against MM cells. The α-T3E-dependent disruption of the homeostasis of proteasomes depended on decreases in proteasome subunits via the inactivation of signal transducer and activator of transcription 3 (STAT3) and nuclear factor erythroid 2 related factor-1 (NRF1), which inhibited protease activity, such as chymotrypsin-like activity, in proteasomes. The α-T3E-dependent inhibition of this activity also induced severe ER stress and ultimately resulted in effective cytotoxicity against MM cells with chemoresistance. The present results indicate that α-T3E acts as an effective anti-mesothelioma agent by disrupting the homeostasis of proteasomes through the simultaneous inactivation of STAT3 and NRF1.

## 1. Introduction

Malignant mesothelioma (MM) is a highly aggressive tumor type that is typically caused by exposure to asbestos and has limited treatment options [1]. Chemotherapy resistance in cancer cells continues to be a major clinical obstacle to the successful treatment of cancer. MM is highly chemoresistant and, thus, has a poor prognosis, with median survival ranging between 8 and 14 months after diagnosis [2]. With this unmet clinical need, the development of new effective anti-MM agents and MM therapeutic strategies is required to improve the treatment of MM.

There are currently many therapeutic targets for the treatment of cancer, and proteasomes are considered to be promising [3]. The proteasome system is the main protein disposal system in cells for the degradation of denatured proteins, and the proteasome-mediated hydrolysis of more than 80% of all cellular proteins determines their cell survival [3]. Proteasome inhibitors, such as carfilzomib, are utilized to treat multiple myeloma and effectively induce cell death in cancer cells [3,4]; therefore, proteasomes are a promising target in the establishment of effective cancer treatment strategies. Moreover, a recent study revealed the overexpression of proteasomal subunits in MM tissues [5], indicating the potential of proteasomes as a target in the treatment of MM. Signal transducer and activator of transcription 3 (STAT3) and nuclear factor erythroid 2 related factor-1 (NRF1) play central roles in maintaining the homeostasis of proteasomes in cancer cells [6,7]. STAT3 is persistently activated in cancer cells and functions as a positive transcription factor to induce the expression of various pro-survival genes that play roles in oncogenic processes [8,9]. Furthermore, proteasomal subunits are involved in STAT3-targeted molecules, with the inhibition of STAT3 downregulating the expression of these subunits [6]. NRF1 also acts as a positive transcription factor that induces proteasomal subunits [7]. When proteasome activity is sufficient, NRF1 is generally degraded by proteasomes [10,11,12]. However, when the functions of proteasomes are impaired, NRF1 may be activated into a mature transcription factor located in the nucleus that induces the expression of proteasome genes [10,11,12]. This process is called the bounce-back response of proteasomes, which has been shown to undermine proteasome inhibitor cytotoxicity in cancer cell lines [10,11,12]. Based on these findings, STAT3 and NRF1 have been proposed as the most important transcription factors for maintaining the homeostasis of proteasomes in cancer cells.

We have already reported that a redox-silent analogue of tocotrienol, 6-*O*-carboxypropyl-alpha-tocotrienol (α-T3E), effectively induces cell death in MM cells without a negative impact on non-tumor cells and also that α-T3E-mediated cytotoxicity against MM cells was partly dependent on the inactivation of STAT3 [8]. These findings indicated that α-T3E disrupted the homeostasis of proteasomes by inactivating STAT3. Furthermore, the effects of α-T3E on NRF1 currently remain unclear. Collectively, it is likely that α-T3E-induced cytotoxicity against MM cells might depend on the disruption of homeostasis in proteasomes via the simultaneous inactivation of STAT3 and NRF1. Thus, this study was undertaken to clarify this possibility.

## 2. Results

### 2.1. α-T3E Induces Cell Death in Human MM Cells

**Figure 1:**
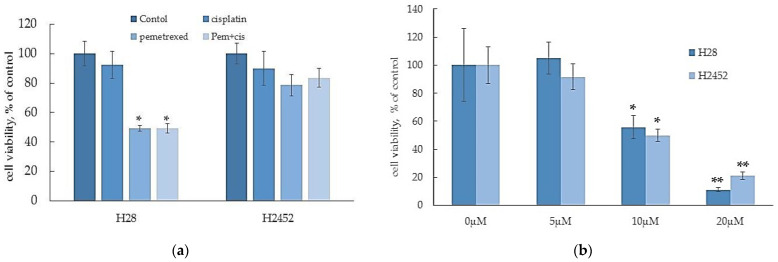
Effects of anti-cancer agents (**a**) and α-T3E (**b**) on the viabilities of H2452 cells and H28 cells. Cells were treated with cisplatin (5 μM), pemetrexed (2.5 μM), a combination of cisplatin (5 μM) and pemetrexed (2.5 μM), and α-T3E (0–20 μM) for 24 h, and each cell viability was evaluated by the WST-8 assay as described in Section 4.4. The data are means ± SD, *n* = 5. * *p* < 0.05, ** *p* < 0.01 vs. the control.

A recent study demonstrates that MM cells have strong chemoresistance against several types of anti-cancer agents [13]. Therefore, we initially examined chemoresistance in H28 cells and H2452 cells. As shown in Figure 1a, in comparisons with the control treatment, cisplatin, pemetrexed, and their combination induced approximately 20, 50, and 50% reductions, respectively, in the viability of H28 cells; however, the same treatments did not affect the viability of H2452 cells. On the other hand, α-T3E exhibited effective cytotoxicity against the two MM cell lines in a dose-dependent manner, with 20 µM α-T3E inducing more than 80% decreases in the viabilities of these MM cell lines (Figure 1b). Since this treatment dose of α-T3E is within the maximum level in the plasma of mice treated with an adequate dose of α-T3E, which had no adverse effects and inhibited the in vivo growth of MM cells [14], α-T3E may function as an effective anti-mesothelioma agent in MM cells with strong chemoresistance.

### 2.2. α-T3E Inhibits Proteasome Activity in Human MM Cells

**Figure 2:**
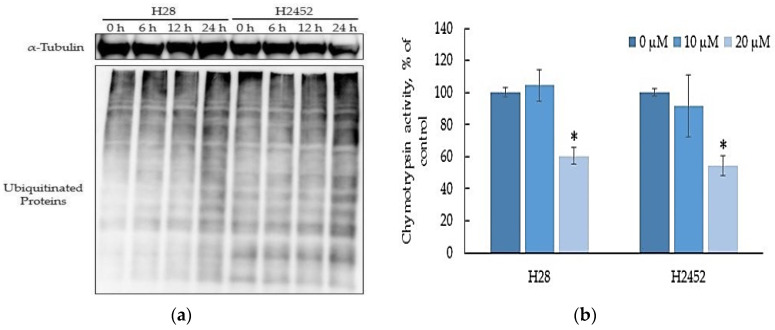
Effects of α-T3E on proteasome activities in H2452 cells and H28 cells. (**a**) Cells were treated with 20 µM α-T3E for 0, 6, 12, or 24 h. After the treatment, the ubiquitinated protein levels in the samples were assessed by immunoblotting as described in Section 4.7. The α-Tubulin protein levels served as the loading control. The results are representative of two independent experiments. (**b**) After cells were treated with α-T3E (10–20 μM) for 24 h, chymotrypsin-like activity was assessed by a chemiluminescent method as described in Section 4.5. The data are means ± SD, *n* = 3. * *p* < 0.05 vs. the control.

Since it is a subtype of vitamin E, δ-tocotrienol has been shown to exert inhibitory effects on proteasome activity in cancer cells, which contributed to its cytotoxicity [15], it is hypothesized that α-T3E may also act as an effective inhibitor against proteasomes in MM cells. A typical marker of the inhibition of proteasome activity, polyubiquitinated protein levels in H2452 cells and H28 cells, showed time-dependent increases after the α-T3E treatment (Figure 2a). Consistent with this result, 20 µM α-T3E significantly inhibited chymotrypsin-like activity in the proteasomes of MM cells (Figure 2b). These results indicated that α-T3E functioned as a proteasome inhibitor in MM cells.

### 2.3. The Inactivation of STAT3 by α-T3E Contributes to the Inhibition of Proteasome Activity in Human MM Cells

**Figure 3:**
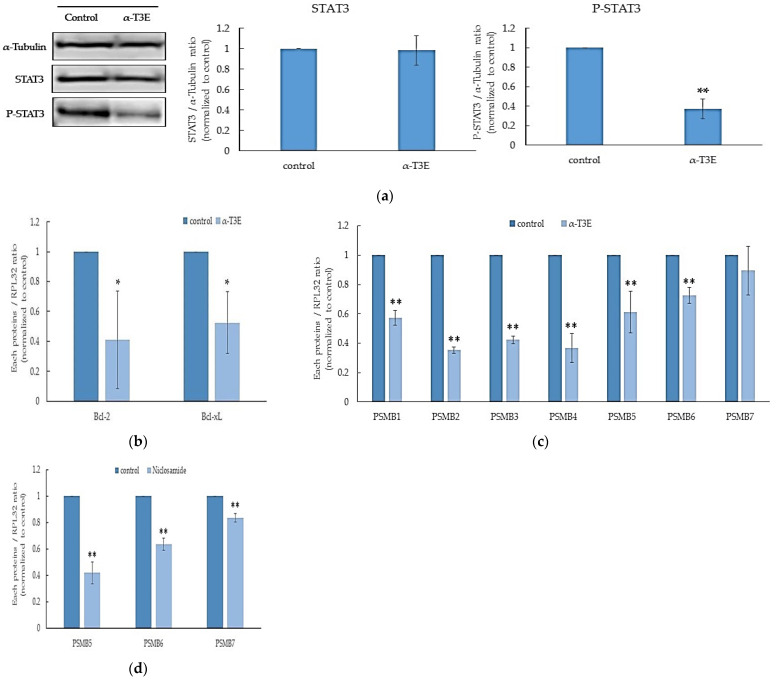
Contribution of α-T3E-dependent STAT3 inactivation to the inhibition of proteasome subunits in H2452 cells. (**a**) Cells were treated with α-T3E (20µM) for 24 h, and STAT3 and phosphorylated P-STAT3 protein levels were assessed by immunoblotting. The α-Tubulin protein levels served as the loading control. A densitometric analysis was performed as described in Section 4.7. The data are means ± SD, *n* = 3. ** *p* < 0.01 vs. the control. (**b**) Cells were treated with α-T3E (20 µM) for 24 h, and the mRNA levels of BCL-2 and BCL-XL were assessed by real-time quantitative PCR as described in Section 4.6. The data are means ± SD, *n* = 3. * *p* < 0.05 vs. the control. (**c**) Cells were treated with α-T3E (20 µM) for 24 h, and the mRNA levels of proteasome subunits were assessed by real-time quantitative PCR. The data are means ± SD, *n* = 3. ** *p* < 0.01 vs. the control. (**d**) Cells were treated with niclosamide (10 µM) for 24 h, and the mRNA levels of proteasome subunits were assessed by real-time quantitative PCR. The data are means ± SD, *n* = 3. ** *p* < 0.01 vs. the control.

A recent study reports that STAT3 acts as a positive transcription factor to induce the expression of the main parts of proteasome subunits in cancer cells [6]. Furthermore, we have already demonstrated that α-T3E inhibits the activation (phosphorylation) of STAT3 in human MM cells [8]. Based on these findings, we speculated that α-T3E inhibited the functions of proteasomes via the STAT3-inactivation-dependent suppression of proteasome subunits and confirmed that this was the case in the present study. As shown in Figure 3a, the α-T3E treatment significantly inhibited the activation (phosphorylation) of STAT3 in H2452 cells. Consistent with this result, target molecules of STAT3, B cell lymphoma (BCL)-2, and BCL-xL [16] were significantly suppressed at the mRNA level by the α-T3E treatment (Figure 3b). Moreover, the α-T3E treatment significantly inhibited the expression of the STAT3-targeted molecules, proteasome subunit beta type (PSMB) 1–6 (the main subunits of proteasomes), and slightly suppressed that of PSMB7. These results suggested that α-T3E functioned as a proteasome inhibitor by inactivating STAT3, which was supported by the result showing that the STAT3 inhibitor, niclosamide (positive control) significantly reduced the mRNA levels of PSMB 5–7 (Figure 3d).

### 2.4. α-T3E-Dependent Inactivation of NRF1 Functions Contributes to the α-T3E-Dependent Inhibition of Proteasome Functions in Human MM Cells

**Figure 4:**
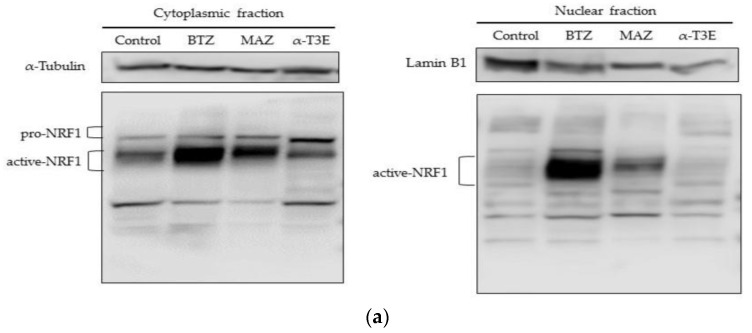

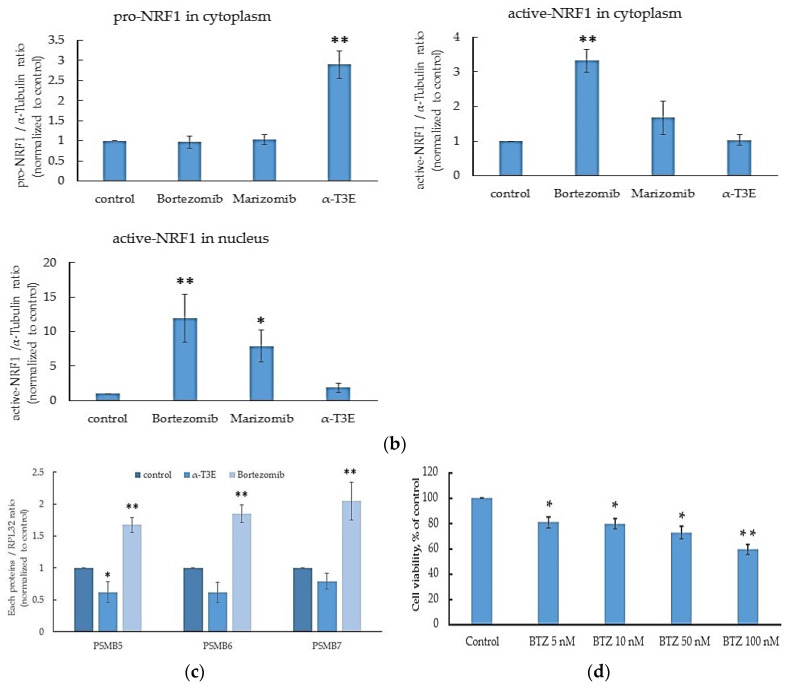
Inhibitory effects of α-T3E on NRF1 activation in H2452 cells. (**a**) Cells were treated with α-T3E (20 µM), bortezomib (50 nM), and marizomib (10 nM) for 12 h, and the protein levels of pro-NRF1 and active NRF1 in the cytoplasm and active NRF1 in the nucleus were assessed by immunoblotting. The α-Tubulin protein levels and lamin B1 protein levels served as the loading controls in the cytoplasm and nucleus, respectively. These results are representative of three independent experiments. (**b**) A densitometric analysis of pro-NRF1 and active NRF1 in the cytoplasm and active NRF1 in the nucleus was performed as described in Section 4.7. The data are means ± SD, *n* = 3. * *p* < 0.05, ** *p* < 0.01 vs. the control. (**c**) Cells were treated with α-T3E (20 µM) and bortezomib (50 nM) for 12 h, and the mRNA levels of proteasome subunits were assessed by real-time quantitative PCR. The data are means ± SD, *n* = 3. * *p* < 0.05, ** *p* < 0.01 vs. the control. (**d**) Cells were treated with each concentration of bortezomib for 24 h, and cell viability was assessed by WST-8. The data are means ± SD, *n* = 5. * *p* < 0.05, ** *p* < 0.01 vs. the control.

A recent study reports that pro-NRF1 (the inactive form of NRF1) is cleaved to active NRF1 via processing under the inhibition of proteasomes and also that active NRF1 functions as a positive transcription factor to induce the expression of the main proteasome subunits [12]. Additionally, the activation of NRF1 under these conditions may compensate for the inhibition of proteasomes [11]. Based on these findings, we investigated whether the activation of NRF1 under the α-T3E-dependent inhibition of proteasomes was suppressed in H2452 cells. As shown in Figure 4a,b, the cytoplasmic and nuclear levels of the active NRF1 protein were significantly higher in H2452 cells treated with the proteasome inhibitors bortezomib and marizomib than in those of the control. On the other hand, no significant differences were observed in the cytoplasmic or nuclear levels of the active NRF1 protein in H2452 cells treated with α-T3E and in those of the control (Figure 4a,b). Furthermore, the cytoplasmic levels of the pro-NRF1 protein were significantly higher in H2452 cells treated with α-T3E than in the three other groups examined (Figure 4a,b). As shown in Figure 4c, bortezomib significantly increased the mRNA levels of PSMB5-7, which might be upregulated by NRF1, whereas α-T3E significantly decreased PSMB5-7 mRNA levels. Additionally, in comparison with the control, a high dose of bortezomib (100 nM) as a positive control induced an approximately 40% decrease in cell viability (Figure 4d); however, this effect of bortezomib was markedly weaker than that of α-T3E at a pharmacological dose (20 µM), as shown in Figure 1b. Therefore, α-T3E may function as an effective proteasome inhibitor by attenuating the activation of NRF1 under the suppression of proteasomes, in contrast to other proteasome inhibitors.

### 2.5. α-T3E Induces ER Stress, Leading to Cell Death in Human MM Cells

**Figure 5:**
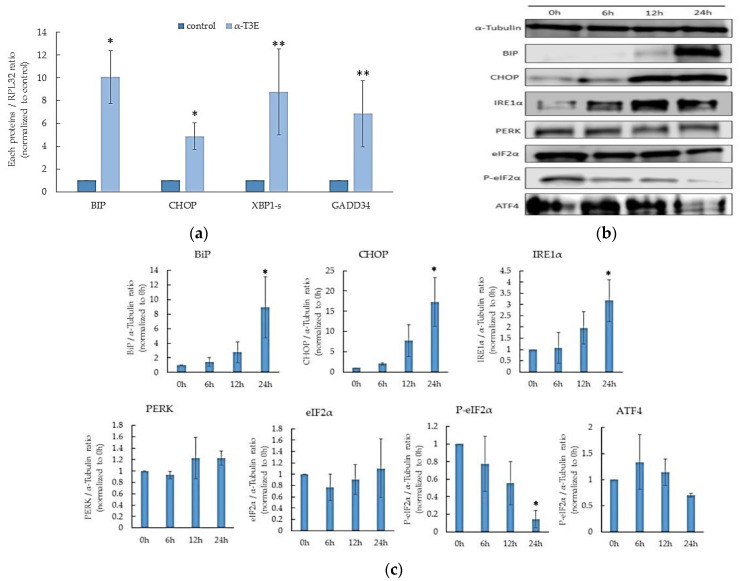
Inducing effects of α-T3E on ER stress in H2452 cells. (**a**) Cells were treated with α-T3E (20 µM) for 24 h, and the mRNA levels of ER-stress-related molecules were assessed by real-time quantitative PCR. The data are means ± SD, *n* = 3. * *p* < 0.05, ** *p* < 0.01 vs. the control. (**b**) Cells were treated with α-T3E (20 µM) for the indicated periods, and the levels of ER-stress-related proteins were measured by immunoblotting. The α-Tubulin protein levels served as the loading control. (**c**) A densitometric analysis of ER-stress-related proteins was performed. The data are means ± SD, *n* = 3. * *p* < 0.05 vs. the control.

Recent studies demonstrate that the inhibition of proteasome functions in cancer cells strongly induces endoplasmic reticulum (ER) stress due to the marked accumulation of denatured proteins in the ER [17,18]. The present results also showed the inhibitory effects of α-T3E on proteasomal activity in human MM cells. Therefore, α-T3E may induce ER stress, which leads to cell death in human MM cells. Based on this speculation, we investigated whether α-T3E induced ER stress and, ultimately, cell death [19] in H2452 cells. As shown in Figure 5a, the mRNA levels of the ER-stress-induced cell-death-related molecules [19], binding immunoglobulin protein (BIP), C/EBP homologous protein (CHOP), spliced-X-box-binding protein-1 (sXBP1), and growth arrest and DNA damage-inducible protein 34 (GADD34), were significantly increased by the α-T3E treatment. Consistent with this result, the protein levels of BIP, CHOP, and inositol-requiring enzyme-1α (IRE1α) were elevated by the same treatment (Figure 5b). Increases in the mean ratio of each protein level in the α-T3E-treated group for 24 h/0 h group (*n* = 3) were as follows: BIP, 8.92-fold; CHOP, 17.20-fold; and IRE1α, 3.17-fold. In contrast, the α-T3E treatment inhibited the protein kinase-like ER kinase/eukaryotic initiation factor 2 α (eIF2α)/activating transcription factor 4 (ATF4) signal pathway related to ER-stress-mediated cell survival [20] (Figure 5b). Increases in the mean ratios of phosphorylated-eIF2α/eIF2α and ATF4 in the α-T3E-treated group for 24 h/0 h group (*n* = 3) were 0.047-fold and 0.81-fold, respectively. Overall, these results suggested that the α-T3E treatment strongly induced ER stress in H2452 cells via the inhibition of proteasomes.

## 3. Discussion

We have already have already reported that α-T3E functioned as an effective anti-cancer agent in some cancer cells, including MM cells, which have strong chemoresistance [21,22]. However, the mechanisms by which α-T3E exhibits effective cytotoxicity against refractory cancer cells currently remain unclear. On the other hand, since the functions of proteasomes are found to be enhanced in MM cells, the abrogation of this enhancement may be a key factor for overcoming the chemoresistance of these cells [5]. To elucidate the underlying mechanisms, we herein investigated whether α-T3E exhibited cytotoxicity against MM cells by inhibiting the functions of proteasomes.

The combination of cisplatin and pemetrexed is generally performed as standard chemotherapy for MM patients; however, it is not curative [23]. In the present study, this combination was moderately effective against H28 cells, but not H2452 cells, which confirmed that it is ineffective as chemotherapy for MM patients. Nevertheless, we also showed that irrespective of the differences in sensitivity to this combination treatment between H28 cells and H2452 cells, α-T3E (20 µM) at a pharmacological dose induced similar decreases in the viabilities of these two cell lines. Since we previously confirmed that this α-T3E treatment dose had no adverse effects on non-tumorigenic cells [8], α-T3E is a promising anti-MM agent with less toxicity.

A previous recent study demonstraets that STAT3 functions as a positive transcription factor to induce the expression of proteasomal subunits and that the inhibition of STAT3 is closely associated with decreases in the expression levels of proteasome subunits and proteasome-mediated protease activity in prostate cancer [6]. In the present study, we confirmed that α-T3E significantly inhibited the activation (phosphorylation) of STAT3, and this result was consistent with our previous findings [8]. As expected, we found that α-T3E significantly suppressed the expression of proteasome subunits (PSMB) and proteasomal chymotrypsin-like activity in H2452 cells. We also demonstrated that the inhibition of STAT3 induced the suppression of PSMB in H2452 cells using a STAT3 inhibitor (niclosamide). Collectively, the findings and the present results revealed that α-T3E disrupted the homeostasis of proteasomes in H2452 cells through the inactivation of STAT3.

MM tissues have been shown to overexpress proteasomes; however, bortezomib is ineffective as an anti-mesothelioma agent to treat MM patients [5]. These findings suggest that the suppression of proteasomes by proteasome inhibitors is insufficient as an effective MM therapy due to the NRF1-mediated recovery of proteasomes, called the bounce-back response of proteasome, in MM tissues [10,11,12]. In the present study, we confirmed the NRF1-mediated bounce-back response in MM cells. We showed that bortezomib and marizomib markedly increased active NRF1 protein levels in the cytoplasm and nucleus of MM cells as well as the NRF1-targeted levels of proteasome subunits. Additionally, the pharmacological plasma levels of bortezomib without severe side effects (5–50 nM) did not induce cell death in MM cells. In contrast, although α-T3E exerted inhibitory effects on the functions of proteasomes, it did not increase the active NRF1 protein levels in the cytoplasm or nucleus or the NRF1-targeted levels of PSMB in MM cells. These results clearly demonstrated the ability of α-T3E to suppress the NRF1-mediated bounce-back response in MM cells, irrespective of the agent-mediated inhibition of proteasome functions, thereby contributing to effective cytotoxicity against MM cells.

NRF1 generally exists in the ER in its N-glycosylated form, is activated by processing in which the glycosylated form is de-glycosylated by N-glycanase 1 (NGLY1) and is proteolytically cleaved by the aspartyl protease DNA damage inducible 1 homolog 2 (DDI2) [24,25]. These findings also suggest that the inhibition of NGLY1 and silencing of DDI2 in cancer cells effectively suppress the formation of the active NRF1 protein as well as the bounce-back response in proteasomes. In the present study, α-T3E promoted the accumulation of the pro-NRF1 form in the cytoplasm of MM cells, indicating that it suppressed the bounce-back response in the proteasomes of MM cells by inhibiting the processing of the pro-NRF1 form by NGLY and DDI2. However, further studies are needed to clarify this speculation.

Recent studies have reported that in case of the accumulation of denatured proteins in the ER of cancer cells, the unfolded protein response (UPR) occurs, and that, as a result, the levels of CHOP related to cell death caused by ER stress, BIP, a chaperone in ER, and IRE1α and sXBP1 located upstream of CHOP and BIP, are elevated [19,26]. Furthermore, protein levels in the ER are tightly regulated by proteasome-mediated ER-associated degradation [27], and proteasome inhibitors may induce ER stress in cancer cells [28]. In the present study, we showed that α-T3E increased the mRNA levels of BIP, CHOP, sXBP1, and GADD34, as well as the protein levels of BIP, CHOP, and IRE1α in MM cells, indicating that α-T3E induced ER stress in MM cells via the effective inhibition of proteasome functions. The PEAK/eIF2α/ATF4 signal pathway has dual roles that contribute to cell death or survival in cancer cells depending on the variable conditions of the ER in cancer cells [24,29]. The present results suggested that α-T3E induced cell death irrespective of the inactivation of the PEAK/eIF2α/ATF4 signal pathway. Thus, the α-T3E-induced inactivation of the PEAK/eIF2α/ATF4 signal pathway observed in the present study appeared to contribute to cell death via the suppression of signals related to cell survival. The mechanisms by which α-T3E inactivates the PEAK/eIF2α/ATF4 signal pathway currently remain unclear. However, a recent study reports that the induction of ER stress in cancer cells activates the PEAK/eIF2α/ATF4 signal pathway and that GADD34 induced by severe or chronic ER stress inhibits the PEAK/eIF2α/ATF4 signal pathway via the de-phosphorylation of eIF2α [26,30]. In the present study, α-T3E increased the expression level of GADD34 in MM cells. Therefore, the increases in GADD34 levels caused by α-T3E-induced severe ER stress on MM cells appear to be closely associated with the inactivation of the PEAK/eIF2α/ATF4 signal pathway.

In summary, α-T3E disrupted the homeostasis of proteasomes in MM cells with severe chemoresistance via the simultaneous inactivation of STAT3 and NRF1, leading to the induction of severe ER stress and the subsequent cell death. Collectively, it seems to be possible that α-T3E is a promising candidate to establish a new MM treatment strategy. To clarify this possibility, a clinical trial in MM patients using α-T3E is absolutely needed.

## 4. Materials and Methods

### 4.1. Reagents

All cultures and chemicals were purchased from Nacalai Tesque (Kyoto, Japan), unless otherwise indicated. Fetal bovine serum (FBS) was purchased from Bio West (Nuaillé, France). Cisplatin, pemetrexed, and bortezomib (a protease inhibitor) were obtained from Wako Chemicals (Osaka, Japan). Niclosamide (a STAT3 inhibitor) and marizomib (a proteasome inhibitor) were purchased from Abcam (Cambridge, UK) and MedChem Express (Monmouth Junction, NJ, USA), respectively. T3 was purchased from Tama Biochemicals (Tokyo, Japan). All antibodies, except for NRF1 and Lamin B1, were obtained from Cell Signaling Technology, Inc. (Beverly, MA, USA). Antibodies for NRF1 and Lamin B1 were purchased from ATLAS Antibodies (Stockholm, Sweden) and ProteinTech (Tokyo, Japan), respectively.

### 4.2. α-T3E Synthesis

α-T3E was synthesized from T3 according to a previously reported procedure [22]. The purity of α-T3E was confirmed by GC–MS, 1H NMR, 13C NMR, and IR. The NMR and IR spectra were consistent with the structure of α-T3E. 1H NMR (CDCl3) spectrum: 1.27 (3H, s), 1.59 (9H, s), 1.67 (3H, s), 2.00 (3H, s), 2.09 (3H, s), 2.12 (3H, s), 1.70–2.15 (16H, m), 2.57 (2H, t, J = 7.8 Hz), 2.65 (2H, t, J = 6.5 Hz), 3.68 (2H, t, J = 7.7 Hz), 4.95–5.25 (3H, m), and 8.5 (1H, broad). IR (KBr) spectrum: 3200–3400 cm^−1^ (carboxylic OH) and 1710 cm^−1^ (C6=O).

### 4.3. Cell Culture

H28 cells and H2452 cells (ATCC, Manassas, VA, USA) were routinely grown in RPMI1640 supplemented with 10% FBS, 6.5 mg/mL glucose, 1 mM sodium pyruvate, 10 mM HEPES, 50 IU/mL penicillin, and 50 μg/mL streptomycin at 37 °C in a humidified atmosphere with 5% CO_2_. Exponentially growing cells were used in experiments. Cells were plated on culture plates and cultured for 24 h to permit adherence. Cells were then cultured in RPMI1640 supplemented with 2%FBS, 6.5 mg/mL glucose, 1 mM sodium pyruvate, and 10 mM HEPES containing each reagent for the indicated period, and each parameter was then examined.

### 4.4. Cell Viability

The WST-8 assay was performed to evaluate the effects of each reagent on the viability of H28 cells and H2452 cells. Cells were seeded on a 96-well plate (5 × 10^3^ cells/well), cultured for 24 h, and were subsequently treated with each reagent (α-T3E, cisplatin, pemetrexed, cisplatin plus pemetrexed, and bortezomib) for 24 h, as described in each figure legend. After each treatment, 10 μL of WST-8 solution was applied to each well containing 100 μL of the cell suspension and was incubated at 37 °C for a further 30 min in 5% CO_2_. The color development was monitored at 450 nm using a multi-well plate reader (SUNRISE Rainbow RC-R, Tecan Japan, Kanagawa, Japan).

### 4.5. Proteasome Activity

H2452 cells were seeded on a 96-well white plate (5 × 10^3^ cells/100 µL/well), cultured for 24 h, and subsequently treated with α-T3E (20 µM) or vehicle for 24 h. After the treatment, to assess the chymotrypsin-like activity in cells, 50 µL of Proteasome-Glo™ Chymotrypsin-Like Cell-Based Assay Reagent (Promega Japan, Tokyo, Japan) was added to each well, and the plate was then incubated at room temperature for 20 min. The chymotrypsin-like activity was assessed based on the estimated chemiluminescence intensity using a luminometer (Infinite M1000 PRO, TECAN Japan).

### 4.6. Isolation of mRNA and Real-Time Quantitative PCR

H2452 cells were cultured at a density of 5 × 10^5^ cells in a 60 mm dish for 24 h, and were then treated with each agent (α-T3E and niclosamide) for 12–24 h. After the treatment, cells were collected, and total RNA was isolated using the Tissue Total RNA Extraction Mini Kit (Favorgen Biotech Corp., Ping-Tung, Taiwan). Total RNA (300 ng for each sample) was used for cDNA synthesis with the ReverTra Ace qPCR RT Kit (Toyobo, Osaka, Japan). cDNA templates were analyzed by real-time PCR using a Thermal Cycler Dice Real Time System Lite (TAKARA BIO INC., Shiga, Japan) and THUNDER-BIRD™ SYBR qPCR Mix (Toyobo, Osaka, Japan) with the following program: at 95 °C for 10 s, followed by 40 cycles at 95 °C for 15 s and at 60 °C for 1 min. The primer sets are shown in Table 1. The gene expression data were normalized to the expression of the reference gene ribosomal protein L32.

### 4.7. Immunoblotting

H2452 cells were cultured at a density of 5 × 10^5^ cells in a 60 mm dish for 24 h and then treated with each agent (α-T3E, bortezomib, and marizomib) for 12–24 h. After the treatment, cells were harvested and lysed in ice-cold Laemmli sample buffer (Bio-Rad, Berkeley, CA, USA) containing protease inhibitor cocktail (Nakalai Tesque, Kyoto, Japan) and phosphatase inhibitor (Nacalai Tesque). Cells were incubated on ice for 20 min following centrifugation at 12,000 rpm at 4 °C for 10 min. The samples were electrophoresed through a 10% or 15% SDS–polyacrylamide gel and transferred to a polyvinylidene difluoride membrane using the iBlot 2 Dry Blotting System (Thermo Fisher Scientific, Waltham, MA, USA). Membranes were blocked with Blocking One P (Nacalai Tesque) for 1 h, incubated with primary antibodies for 1 h, and then incubated with the secondary antibody for 1 h. Detection was accomplished using Chemi-Lumi One Super (Nacalai Tesque) and C-DiGit (LI-COR, Lincoln, NE, USA). A densiometric analysis of each immune band was performed using Image Studio for C-DiGit (LI-COR). Molecular sizing was conducted using Rainbow MW markers (Amersham Japan, Tokyo, Japan). Protein concentrations were assessed using the DC Protein Assay System (Bio-Rad).

### 4.8. Statistical Analysis

Differences among groups were analyzed by a one-way ANOVA followed by the Tukey–Kramer test, while differences between two groups were examined by a one-way ANOVA followed by Student’s *t*-test. All statistical analyses were performed using Ekuseru-Toukei software (Social Survey Research Information Co., Ltd., Tokyo, Japan). Differences with *p*-values of 0.05 or less were considered to be significant. All experiments were conducted with a minimum of three samples from three independent experiments, and the data were expressed as means ± SD. The number of samples in each experiment is shown in the respective figure legends.

## Figures and Tables

**Table 1:** List of PCR primers.

Gene	Primer	Sequence
BCL-2	Forward Primer	GTGTGTGGAGAGCGTCAACC
	Reverse Primer	CAGCCAGGAGAAATCAAACAGA
BCL-XL	Forward Primer	ACCTGACATCCCAGCTCCAC
	Reverse Primer	GTCTACGCTTTCCACGCACA
PSMB1	Forward Primer	TTTCGCCCTACGTTTTCAAC
	Reverse Primer	TACAGCCCCCTTTCCTTCTT
PSMB2	Forward Primer	AAGGCCCCGACTATGTTCTT
	Reverse Primer	AGGTTGGCAGATTCAGGATG
PSMB3	Forward Primer	GAAGGGGAAGAACTGTGTGG
	Reverse Primer	CCTGGTGGTGATTTTGTCCT
PSMB4	Forward Primer	TCAGTCCTCGGCGTTAAGTT
	Reverse Primer	GCTTAGCACTGGCTGCTTCT
PSMB5	Forward Primer	CCATACCTGCTAGGCACCAT
	Reverse Primer	GCACCTCCTGAGTAGGCATC
PSMB6	Forward Primer	CCTATTCACGACCGCATTTT
	Reverse Primer	TCCCGGTAGGTAGCATCAAC
PSMB7	Forward Primer	CGGCTGTGTCGGTGTATG
	Reverse Primer	GCCAGTTTTCCGGACCTT
BIP	Forward Primer	CGAGGAGGAGGACAAGAAGG
	Reverse Primer	CACCTTGAACGGCAAGAACT
CHOP	Forward Primer	GGAGGTGGAAGCCTGGTATG
	Reverse Primer	GTGACCTCTGCTGGTTCTGG
s-XBP1	Forward Primer	GGTCTGCTGAGTCCGCAGCAGG
	Reverse Primer	GGGCTTGGTATATATGTGG
GADD34	Forward Primer	GAAGGAGGAAAAGGCACACA
	Reverse Primer	CCTCACCCTCCTCTTCATCAC
RPL32	Forward Primer	AACCCTGTTGTCAATGCCTC
	Reverse Primer	CATCTCCTTCTCGGCATCA

## Data Availability

All relevant data are within the manuscript.
